# Clinical outcomes of percutaneous coronary intervention for *de novo* lesions in small coronary arteries: A systematic review and network meta-analysis

**DOI:** 10.3389/fcvm.2022.1017833

**Published:** 2022-11-14

**Authors:** Wen-Rui Ma, Karthik H. Chandrasekharan, Chang-Sheng Nai, Yong-Xiang Zhu, Javaid Iqbal, Shang Chang, You-Wei Cheng, Xin-Yu Wang, Christos V. Bourantas, Yao-Jun Zhang

**Affiliations:** ^1^Department of Cardiology, Xuzhou Third People’s Hospital, Xuzhou Medical University, Xuzhou, China; ^2^Department of Cardiology, Barts Heart Centre, Barts Health NHS Trust, London, United Kingdom; ^3^Department of Cardiology, Sheffield Teaching Hospitals NHS Foundation Trust, Sheffield, United Kingdom

**Keywords:** clinical outcome, *de novo* lesions, drug-coated balloon, new-generation drug-eluting stent, small coronary arteries

## Abstract

**Background:**

Percutaneous coronary intervention (PCI) has a well-established role in revascularization for coronary artery disease. We performed network meta-analysis to provide evidence on optimal intervention strategies for *de novo* lesions in small coronary arteries.

**Materials and methods:**

Enrolled studies were randomized clinical trials that compared different intervention strategies [balloon angioplasty (BA), biolimus-coated balloon (BCB), bare-metal stent (BMS), new-generation drug-eluting stent (New-DES), older generation sirolimus-eluting stent (Old-SES), paclitaxel-coated balloon (PCB), and paclitaxel-eluting stent (PES)] for *de novo* lesions in small coronary arteries. The primary outcome was major adverse cardiac events (MACE).

**Results:**

A total of 23 randomized clinical trials comparing seven intervention devices were analyzed. In terms of the primary outcome, New-DES was the intervention device with the best efficacy [surface under the cumulative ranking curve (SUCRA), 89.1%; mean rank, 1.7], and the Old-SES [risk ratio (RR), 1.09; 95% confidence interval (CI), 0.45–2.64] and PCB (RR, 1.40; 95% CI, 0.72–2.74) secondary to New-DES, but there was no statistically significant difference between these three intervention devices. All DES and PCB were superior to BMS and BA for MACE in both primary and sensitivity analysis. For secondary outcomes, there was no association between all-cause mortality and myocardial infarction (MI) with any intervention strategy, and additionally, the findings of target lesion revascularization (TLR) were similar to the primary outcomes.

**Conclusion:**

Paclitaxel-coated balloon yielded similar outcomes to New-DES for *de novo* lesions in small coronary arteries. Therefore, this network meta-analysis may provide potential support for PCB as a feasible, effective, and safe alternative intervention strategy for the revascularization of small coronary arteries.

**Systematic review registration:**

[https://www.crd.york.ac.uk/PROSPERO/#recordDetails], identifier [CRD42022338433].

## Introduction

Small vessel lesions are commonly observed in patients with coronary stenoses on coronary angiography (1). However, there are no currently available guidelines for the optimal and appropriate intervention strategies for percutaneous revascularization for patients with *de novo* small-vessel coronary disease (2, 3). When compared with treatment with bare-metal stents (BMS), the contemporary intervention strategy with drug-eluting stents (DES), reduces the rates of stent thrombosis from neointimal hyperplasia, but there is some evidence that there is an increased risk of late and very late stent thrombosis (4–6). Furthermore, late complications include in-stent restenosis, and subsequently, the introduction of the drug-coated balloon (DCB) has addressed this (7–9). Despite these advances in treating coronary artery disease, there remains controversy in defining the most appropriate treatment strategy for small-vessel coronary disease.

Paclitaxel and sirolimus and its derivatives are mainly used in coating DES and DCB, and with improving technology and techniques, clinical outcomes varied between the older generation and new-generation DES (New-DES), and between different types of antiproliferative drugs (10, 11). Previous network meta-analysis has suggested that older generation sirolimus-eluting stent (Old-SES) is superior to paclitaxel-eluting stent (PES) and even DCB for target lesion revascularization (TLR) in small-vessel coronary disease (12). However, several recent studies have established that DCB was associated with comparable outcomes for the treatment of *de novo* small-vessel disease when compared with DES (13–15).

Despite the promising outcomes demonstrated by DCB and DES, the most appropriate intervention strategy in terms of clinical outcome remains inconclusive. Therefore, we performed a network meta-analysis comparing the clinical outcomes of the different intervention strategies, with the aim of establishing whether there is one strategy that may be optimal, based on current evidence.

## Materials and methods

This network meta-analysis followed the PRISMA Statement (16) and was registered with PROSPERO (CRD42022338433).

### Data sources and search

PubMed, Embase, and Cochrane databases were systematically searched to collect all eligible randomized clinical trials that assessed different intervention strategies for the treatment of small vessel coronary stenoses up to July 2022. The detailed search strategy is presented in Supplementary Table 1. All citations were imported into Endnote X9 for manual screening according to the inclusion criteria.

### Intervention strategies

The intervention strategies were divided into seven classifications for comparison: balloon angioplasty (BA), biolimus-coated balloon (BCB), BMS, New-DES, Old-SES, paclitaxel-coated balloon (PCB), and PES. Classification of DCB and DES based on the type of antiproliferative drug is included in Supplementary Table 2.

### Study selection

All eligible randomized clinical studies compared intervention devices in *de novo* lesions of native small coronary vessels (vessel diameter ≤2.75 or ≤3.0 mm) and reported one or more clinical outcomes of interest. The primary outcome was major adverse cardiac events (MACE), and secondary outcomes were all-cause mortality, myocardial infarction (MI), and TLR. The definitions of MACE are similar in most studies and are summarized in Supplementary Table 3. Additionally, studies comparing a combination of intervention devices were excluded. Meanwhile, we screened studies from published meta-analyses to compensate for the limitation of the search algorithm.

### Data extraction and quality assessment

Two investigators (WRM, CSN) independently assessed the terms to avoid bias in the data search and abstraction process. The opinion of a third investigator was sought in case of disagreement. Extracted data included study and patient characteristics, the diameter of reference vessels, the longest time of clinical follow-up, and relevant clinical outcomes. The trials were subsequently divided into low risk, unclear risk, and high risk followed by the Cochrane risk of bias assessment tool (17).

### Statistical analysis

We compared different intervention strategies for treating *de novo* lesions in small vessel coronary arteries with a network meta-analysis using a random-effects model in a frequentist framework. All data were analyzed by *network* and *mvmeta* packages using STATA version 13.0 (StataCorp, college station, TX, USA). The risk ratio (RR) and 95% confidence intervals (CIs) were used to evaluate clinical outcomes. We used network plots to visualize the connections between studies in each clinical outcome. The league tables were employed to illustrate the result of direct and indirect comparisons in different clinical outcomes and 95% CI of 1 indicated no statistical significance. The surface under the cumulative ranking curve (SUCRA) was used to rank intervention strategies in clinical outcomes, with larger values ranked relatively higher. The radar plot was used to summarize the ranking of pre-defined clinical outcomes. The heterogeneity was assessed by *I*^2^ and τ^2^ statistics. The node split method was used to detect local inconsistency, and *P*-value > 0.05 indicated no inconsistency. Funnel plots were used to detect the existence of publication bias by visual inspection. Sensitivity analysis was performed on clinical outcomes of MACE and TLR, excluding the PICCOLETO study (18) with a sample size of <100, and was terminated early due to the higher incidence of MACE suffered by the DCB group.

## Results

### Study selection and characteristics

**FIGURE 1:**
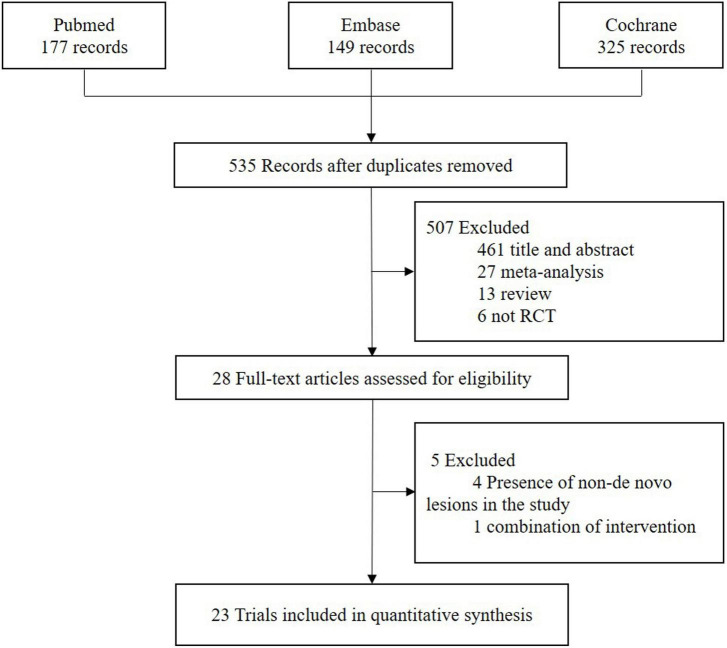
Flowchart of study selection.

**FIGURE 2:**
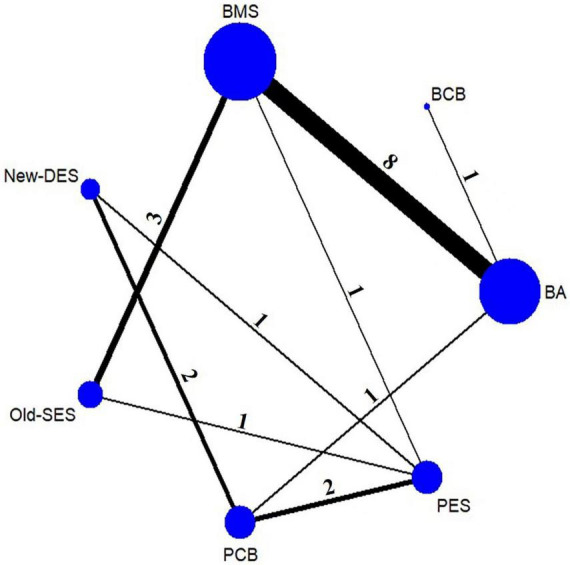
Network plot of intervention strategies for MACE. The nodes indicate intervention strategies. The size of the nodes represents the sample size of the study. The line between nodes is the number of studies for direct comparison. BA, balloon angioplasty; BCB, biolimus-coated balloon; BMS, bare-metal stent; New-DES, new-generation drug-eluting stent; Old-SES, older generation sirolimus-eluting stent; PCB, paclitaxel-coated balloon; PES, paclitaxel-eluting stent.

The flowchart of study selection is presented in Figure 1. Twenty-three eligible randomized clinical trials were screened for this network meta-analysis. The characteristics of these included studies are summarized in Table 1. The longest clinical follow-up period was 24 months. The network plot of direct comparisons in seven intervention strategies (BA, BCB, BMS, New-DES, Old-SES, PCB, and PES) was shown in Figure 2. The BCB is the latest intervention device available, leading to limited comparisons with other devices. A large-scale trial, BASKET SMALL 2, was excluded due to a combination of PES and New-DES in the DES group (19).

**TABLE 1:** Basic characteristics of included studies.

Trial	Year	Reference vessel, mm	Interventions	Sample size	Mean age	Males, (%)	Diabetes, (%)	Prior MI, (%)	Unstable angina (%)	Follow up, months
Ardissino et al. (20)	2004	≤2.75	SES/BMS	129 128	63.6 ± 11.27 63.7 ± 10.9	76.7 66.4	19.4 29.7	29.5 28.1	NA	8
BELLO	2015	<2.8	PCB/PES	90 92	64.8 ± 8.5 66.4 ± 9.0	80.0 77.2	43.3 38.0	51.1 35.9	24.4 21.7	24
BESMART	2001	<3.0	BMS/BA	176 166	62 ± 10 61 ± 10	73.4 79.3	22 12	31.7 43.3	50.0 42.8	6
BIO-RISE CHINA	2022	2.0–2.75	BCB/BA	105 103	61.3 ± 8.8 61.6 ± 8.1	72.4 66.3	34.3 34.7	20.0 26.7	60.0 61.4	12
COAST	2015	2.0–2.6	BMS/BA	393 195	61 ± 10 61 ± 10	72.3 75.1	19.6 17	48.3 46.0	17.5 15	8
C-SIRIUS	2003	2.5–3.0	SES/BMS	50 50	60.3 ± 10.6 60.7 ± 9.1	70 68	24 24	48 42	48 54	9
De luca et al. (22)	2006	≤3.0	BMS/BA	387 411	61 ± 12 61 ± 12	74.2 74.2	12.1 11.7	10.6 8.3	NA	12
E-SIRIUS	2003	2.5–3.0	SES/BMS	175 177	62.0 ± 11.4 62.6 ± 10.3	70 71	19 27	41 43	30 36	9
Funatsu et al. (24)	2017	2.0–2.75	PCB/BA	92 41	68 ± 10 69 ± 11	78 68	48 32	NA	NA	6
Hanekamp et al. (25)	2004	<3.0	BMS/BA	250 246	61 ± 9 61 ± 10	64 71	32 32	17 16	NA	12
ISAR-SMART 3	2006	<2.8	SES/PES	180 180	65.7 ± 10.4 67.4 ± 10.9	75 69	NA	31/29	27 35	12
Kinsara et al. (39)	2003	≤2.5	BMS/BA	96 106	54 ± 11 56 ± 11	70 86	61 50	64 59	15 16	6
LASMAL I	2005	<2.9	BMS/BA	124 122	NA	82 73	25 28	NA	65 59	9
LASMAL II	2005	2.0–2.9	BMS/BA	111 109	NA	73 77	NA	NA	64.5 69	12
Park et al. (40)	2000	<3.0	BMS/BA	60 60	60.2 ± 7.5 61.5 ± 8.4	62 65	13.3 11.6	15 10	18.3 20	6
PICCOLETO	2015	≤2.75	PCB/PES	28 29	68 ± 9 67 ± 10	78.6 75.9	37.9 46.4	17.9 20.7	NA	9
PICCOLETO II	2020	2.0–2.75	PCB/EES	118 114	64 ± 23.7 66 ± 23.7	70.3 76.9	38.0 35.4	38 30	14.4 18	12
RESTORE	2018	2.25–2.75	PCB/ZES	116 114	60.1 ± 10.5 60.5 ± 10.8	66.4 77.2	39.7 42.1	22.4 24.6	69.0 71.1	24
SISA	2001	2.3–2.9	BMS/BA	182 169	60.6 ± 10.3 59.9 ± 10.5	66.3 67.0	17.8 20.9	31.9 35.1	34.3 29.1	6
SISCA	2001	2.1–3.0	BMS/BA	74 71	63.1 ± 11.2 62.7 ± 10.1	56.8 73.3	12.2 14.1	41.9 45.1	25.7 21.1	5
SPIRIT III	2009	2.5	EES/PES	160 59	63.84 ± 10.71 63.62 ± 10.31	60.6 47.5	34.4 39.0	23.7 17.2	23.7 31.0	9
STRESS	1998	<3.0	BMS/BA	163 168	59 ± 10 61 ± 11	74 68	17 16	19 15	56 48	12
TAXUS V subgroup	2005	2.25	PES/BMS	106 93	NA	NA	NA	NA	31.5 29.9	9

BA, balloon angioplasty; BMS, bare-metal stent; DCB, drug-coated balloon; EES, everolimus-generation drug-eluting stent; PES, paclitaxel-eluting stent; SES, sirolimus-eluting stent; ZES, zotarolimus-eluting stent.

### Risk of bias assessment

The risk of bias assessment of the 23 included trials is presented in Supplementary Figure 1. Furthermore funnel plots for each clinical outcome to visually show publication bias are presented in Supplementary Figure 2. Loop inconsistency was low for all-cause mortality in the primary analysis, and MACE and TLR in the sensitivity analysis (Supplementary Figure 3). In contrast, loop-specific heterogeneity was relatively higher for other clinical outcomes in the primary analysis (Supplementary Figure 4).

### Primary outcome

**FIGURE 3:**
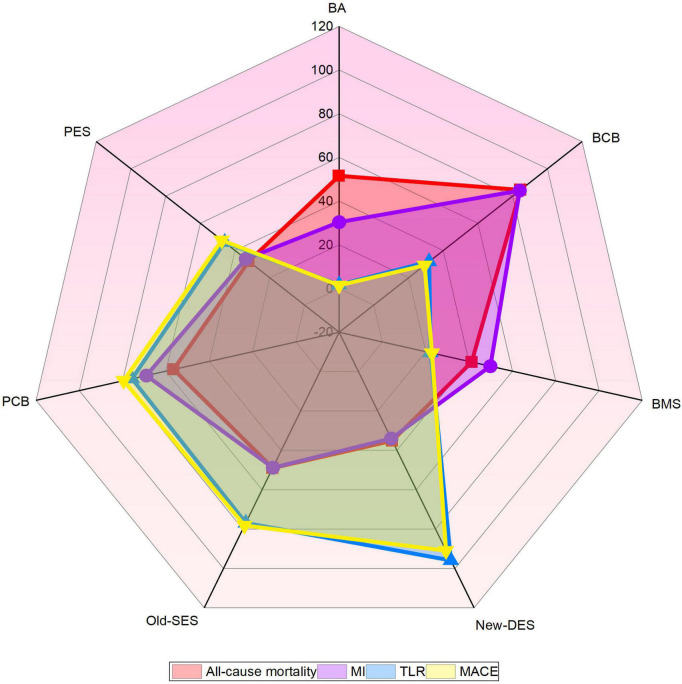
Radar map of ranked intervention strategies in all clinical outcomes. The different colored labeled points represent the SUCRA of each intervention strategy for different clinical outcomes. BA, balloon angioplasty; BCB, biolimus-coated balloon; BMS, bare-metal stent; MACE, major adverse cardiac events; MI, myocardial infarction; New-DES, new-generation drug-eluting stent; Old-SES, older generation sirolimus-eluting stent; PCB, paclitaxel-coated balloon; PES, paclitaxel-eluting stent; TLR, target lesion revascularization.

The network of intervention device comparisons for MACE was available in 20 studies (18, 20–37). All intervention devices except BCB were superior to BMS and with regards to MACE, while BMS was more effective than BA (Table 2A). New-DES was ranked as the most appropriate intervention strategies for MACE (Figure 3), with a non-significant RR of 0.92 (0.38–2.24) compared with Old-SES, 0.71 (0.36–1.40) compared with PCB, 0.53 (0.26–1.09) compared with PES, 0.35 (0.12–1.01) compared with BCB (Table 2A). In addition, Old-SES ranked second to New-DES, with a significant difference RR of 0.57 (0.34–0.98) compared with PES, and 0.38 (0.18–0.81) compared with BCB (Table 2A). For the primary outcome, PCB is more effective than BMS and BA whilst not being inferior to other intervention devices.

**TABLE 2:** The primary network meta-analysis estimates of MACE and TLR.

A						
MACE						
New-DES	1.09 (0.45, 2.64)	1.40 (0.72, 2.74)	1.90 (0.92, 3.92)	2.87 (0.99, 8.27)	3.18 (1.37, 7.38)	4.37 (1.86, 10.28)
0.92 (0.38, 2.24)	Old-SES	1.29 (0.64, 2.58)	1.74 (1.02, 2.98)	2.64 (1.24, 5.62)	2.93 (1.98,4.33)	4.02 (2.63, 6.16)
0.71 (0.36, 1.40)	0.78 (0.39, 1.56)	PCB	1.35 (0.83, 2.20)	2.05 (0.83, 5.03)	2.27 (1.21, 4.27)	3.12 (1.63, 5.96)
0.53 (0.26, 1.09)	0.57 (0.34, 0.98)	0.74 (0.45, 1.20)	PES	1.51 (0.68, 3.35)	1.68 (1.06, 2.66)	2.31 (1.41, 3.78)
0.35 (0.12, 1.01)	0.38 (0.18, 0.81)	0.49 (0.20, 1.20)	0.66 (0.30, 1.46)	BCB	1.11 (0.58, 2.12)	1.52 (0.82, 2.85)
0.31 (0.14, 0.73)	0.34 (0.23, 0.50)	0.44 (0.23, 0.83)	0.60 (0.38, 0.94)	0.90 (0.47, 1.72)	BMS	1.37 (1.16, 1.63)
0.23 (0.10, 0.54)	0.25 (0.16, 0.38)	0.32 (0.17, 0.61)	0.43 (0.26, 0.71)	0.66 (0.35, 1.22)	0.73 (0.61, 0.86)	BA
**B**						
**TLR**						

New-DES	1.20 (0.43, 3.36)	1.96 (0.90, 4.28)	2.55 (1.03, 6.35)	3.92 (0.94, 16.42)	4.97 (1.79, 13.82)	7.48 (2.65, 21.10)
0.83 (0.30, 2.34)	Old-SES	1.64 (0.73, 3.67)	2.13 (1.26, 3.60)	3.28 (1.09, 9.89)	4.15 (2.63, 6.55)	6.24 (3.80, 10.25)
0.51 (0.23, 1.11)	0.61 (0.27, 1.37)	PCB	1.30 (0.68, 2.50)	2.00 (0.56, 7.13)	2.53 (1.15, 5.57)	3.81 (1.70, 8.50)
0.39 (0.16, 0.97)	0.47 (0.28, 0.79)	0.77 (0.40, 1.48)	PES	1.54 (0.49, 4.77)	1.95 (1.15, 3.29)	2.93 (1.68, 5.12)
0.25 (0.06, 1.07)	0.31 (0.10, 0.92)	0.50 (0.14, 1.79)	0.65 (0.21, 2.02)	BCB	1.27 (0.46, 3.47)	1.91 (0.71, 5.11)
0.20 (0.07, 0.56)	0.24 (0.15, 0.38)	0.39 (0.18, 0.87)	0.51 (0.30, 0.87)	0.79 (0.29, 2.16)	BMS	1.50 (1.23, 1.84)
0.13 (0.05, 0.38)	0.16 (0.10, 0.26)	0.26 (0.12, 0.59)	0.34 (0.20, 0.60)	0.52 (0.20, 1.41)	0.67 (0.54, 0.81)	BA

BA, balloon angioplasty; BCB, biolimus-coated balloon; BMS, bare-metal stent; MACE, major adverse cardiac events; New-DES, new-generation drug-eluting stent; Old-SES, older generation sirolimus-eluting stent; PCB, paclitaxel-coated balloon; PES, paclitaxel-eluting stent; TLR, target lesion revascularization. Each area represents the value of RR and 95% CI. The blue area represents different intervention strategies; the brown area represents statistically significant.

### Secondary outcomes

Twenty-three trials yielded data for direct and indirect comparisons of all-cause mortality and MI (18, 20–40). BCB was ranked the highest, followed by PCB (Figure 2), but no statistically significant differences were found among all intervention strategies (Supplementary Table 4).

The results of TLR were similar to those of MACE. New-DES, Old-SES, PCB, and PES had a significantly lower risk of TLR than BMS and BA, whereas BMS was superior to BA (Table 2B). New-DES was the optimal intervention device and Old-SES second best with RR of 0.83 (0.30–2.34) for New-DES vs. Old-SES (Figure 2, Table 2B); followed by PCB with RR of 0.51 (0.23–1.11) for New-DES vs. PCB; by PES with RR of 0.39 (0.16–0.97) for New-DES vs. PES; by BCB with RR of 0.25 (0.06–1.07) for New-DES vs. BCB. Moreover, Old-SES was superior to PES (RR 0.47, 95% CI 0.28–0.79) and BCB (RR 0.31, 95% CI 0.10–0.92) (Table 2B). With regards to TLR, there was no significant difference in the effectiveness of percutaneous coronary intervention (PCI) with PCB device for *de novo* lesions in small coronary vessels vs. DES.

### Sensitivity analysis

A sensitivity analysis was performed for MACE and TLR by excluding PICCOLETO trial due to the potential risk of bias. With regards to MACE, the efficacy of New-DES was superior to both PES (RR 0.42, 95% CI 0.20–0.88) and BCB (RR 0.29, 95% CI 0.10–0.84). Furthermore, PCB was superior to PES (RR 0.53, 95% CI 0.31–0.90) and BCB (RR 0.37, 95% CI 0.15–0.91) for primary outcomes (Table 3A). With regards to TLR, New-DES was superior to BCB (RR of 0.18 95% CI 0.04–0.75); additionally, PCB was superior to BCB (RR 0.44, 95% CI 0.21–0.94) (Table 3B). Similar to the primary analysis, PCB was not inferior to New-DES and Old-SES.

**TABLE 3:** The sensitivity network meta-analysis estimates of MACE and TLR.

A						
MACE						
New-DES	1.25 (0.64, 2.44)	1.32 (0.54, 3.20)	2.37 (1.13, 4.94)	3.41 (1.20, 9.70)	3.84 (1.66, 8.88)	5.20 (2.21, 12.20)
0.80 (0.41, 1.56)	PCB	1.05 (0.51, 2.16)	1.89 (1.11, 3.22)	2.73 (1.10, 6.73)	3.07 (1.59, 5.91)	4.16 (2.13, 8.13)
0.76 (0.31, 1.84)	0.95 (0.46, 1.94)	Old-SES	1.80 (1.07, 3.03)	2.59 (1.24, 5.39)	2.91 (1.99, 4.26)	3.95 (2.61, 5.96)
0.42 (0.20, 0.88)	0.53 (0.31, 0.90)	0.56 (0.33, 0.94)	PES	1.44 (0.67, 3.11)	1.62 (1.04, 2.53)	2.20 (1.37, 3.52)
0.29 (0.10, 0.84)	0.37 (0.15, 0.91)	0.39 (0.19, 0.80)	0.69 (0.32, 1.50)	BCB	1.13 (0.60, 2.11)	1.52 (0.83, 2.79)
0.26 (0.11, 0.60)	0.33 (0.17, 0.63)	0.34 (0.23, 0.50)	0.62 (0.39, 0.96)	0.89 (0.47, 1.66)	BMS	1.35 (1.15, 1.59)
0.19 (0.08, 0.45)	0.24 (0.12, 0.47)	0.25 (0.17, 0.38)	0.46 (0.28, 0.73)	0.66 (0.36, 1.20)	0.74 (0.63, 0.87)	BA
**B**						
**TLR**						

New-DES	1.77 (0.62, 5.09)	1.71 (0.79, 3.73)	3.90 (1.50, 10.11)	5.59 (1.34, 23.44)	7.21 (2.54, 20.45)	10.66 (3.72, 30.60)
0.56 (0.20, 1.62)	Old-SES	0.97 (0.40, 2.33)	2.20 (1.33, 3.65)	3.16 (1.07, 9.32)	4.07 (2.61, 6.34)	6.02 (3.73, 9.72)
0.58 (0.27, 1.27)	1.03 (0.43, 2.49)	PCB	2.27 (1.06, 4.88)	3.26 (0.89, 12.00)	4.20 (1.78, 9.92)	6.22 (2.61, 14.82)
0.26 (0.10, 0.67)	0.45 (0.27, 0.75)	0.44 (0.21, 0.94)	PES	1.44 (0.47, 4.35)	1.85 (1.11, 3.08)	2.74 (1.60, 4.69)
0.18 (0.04, 0.75)	0.32 (0.11, 0.93)	0.31 (0.08, 1.13)	0.70 (0.23, 2.11)	BCB	1.29 (0.48, 3.46)	1.91 (0.72, 5.03)
0.14 (0.05, 0.39)	0.25 (0.16, 0.38)	0.24 (0.10, 0.56)	0.54 (0.32, 0.90)	0.78 (0.29, 2.09)	BMS	1.48 (1.23, 1.78)
0.09 (0.03, 0.27)	0.17 (0.10, 0.27)	0.16 (0.07, 0.38)	0.37 (0.21, 0.63)	0.52 (0.20, 1.38)	0.68 (0.56, 0.81)	BA

BA, balloon angioplasty; BCB, biolimus-coated balloon; BMS, bare-metal stent; MACE, major adverse cardiac events; New-DES, new-generation drug-eluting stent; Old-SES, older generation sirolimus-eluting stent; PCB, paclitaxel-coated balloon; PES, paclitaxel-eluting stent; TLR, target lesion revascularization. Each area represents the value of RR and 95% CI. The blue area represents different intervention strategies; the brown area represents statistically significant.

## Discussion

To our knowledge, this is the first network meta-analysis to compare the efficacy and safety of older generation and new generation PCI devices in the context of small coronary artery stenosis. The major findings are as follows: (1) PCB is not inferior to New-DES and Old-SES in all clinical outcomes of interest and can be considered as an effective alternative intervention device for *de novo* lesions in small coronary arteries; (2) New-DES ranked first but was not statistically different from the Old-SES and PCB either in the primary or sensitivity analysis; (3) There was no significant difference in the risk of all-cause mortality and MI across all included intervention strategies.

A complication of stent implantation is vessel restenosis which can be angiographically quantified measured by late lumen loss, and the absolute value of late lumen loss does not vary by vessel diameter, therefore smaller vessels are more affected by and are more prone to restenosis (41–43). Devices used for PCI are rapidly evolving, with the advent of new-generation devices as described. For instance, DES have been shown to be effective in the treatment of small vessel lesions in multiple studies (26, 34, 35). However, more recently the use of DCB in the treatment of small vessel coronary disease has been highlighted, delivering the highly lipophilic drug directly and rapidly to the vessel wall (43). Specific DCB such as PCB are already established in treating in-stent restenosis (9), and furthermore, DCB are now suggested in guidelines for the treatment of in-stent restenosis, to improve quality of life (44). This study provides further evidence that PCB is as effective as DES for all clinical outcomes of interest in *de novo* lesions of small coronary arteries. Therefore, DCB can be an alternative interventional strategy to DES without implantation.

While this study found that BA without antiproliferative drugs was the least effective intervention device for the treatment of coronary stenoses, PCB combines properties of both BA and DES, i.e., intervening without implantation while delivering antiproliferative drugs to the vessel wall. An additional benefit of PCB compared with DES is the shortened duration of dual antiplatelet therapy, which may be especially valuable in patients with high bleeding risk (45, 46). Moreover, PCB could reduce thrombosis and MI without altering the anatomical morphology of the vessels (47, 48). In addition, consistent with previous studies (37, 49) our additional analysis further provides evidence that DCB was not inferior to DES in terms of TLR and MACE (Supplementary Table 5). Thus, PCB provides effective treatment of *de novo* lesions in small coronary arteries while allowing repeatability of the procedure. In this network meta-analysis, the composite endpoint of MACE mostly consisted of revascularization (Supplementary Table 3), which may drive similar clinical outcomes of MACE and TLR in the treatment of small vessels in *de novo* lesions by different intervention strategies. Alternatively, the pre-determined angiographic follow-up of the included studies may contribute to an increased incidence of TLR, which directly correlates to a higher MACE. Conversely, an observational study reported that the risk of restenosis was twice as high in the DCB group as in the New-DES group. Therefore, the efficacy and safety of DCB still require longer clinical follow-up to be determined (50, 51). In previous studies, the risk of clinical events did not increase significantly over time, and DCB may be an intervention strategy that will benefit patients undergoing PCI in the long term (30, 49). However, the efficacy and safety of DCB still require longer clinical follow-up to be determined. Sirolimus and its derivatives have also been used in DCB in recent years (52, 53), but in this analysis, we could not draw similar conclusions to PCB since only one study included sirolimus-coated balloon was eligible. Similarly, the effect of different brands of DCB varies. The DIOR DCB in the PICCOLETO study was terminated early due to its lower paclitaxel drug concentration resulting in a higher incidence of MACE. However, Venetsanos et al. (54) showed that no significant differences were found among the SeQuent Please, IN.PACT Falcon, and Pantera Lux DCB in clinical outcomes. More studies are needed in the future to demonstrate the availability of sirolimus-coated balloons in small coronary arteries. Compared to older generation DES, the New-DES has improved the technology and technique of stents, contributing to more effective clinical outcomes (55, 56). Recent studies have shown that New-DES could be effective in treating lesions in small coronary arteries (57, 58). New-DES with thinner stent struts may improve the feasibility of the treatment of small coronary arteries. A previous network meta-analysis including 89 trials comparing different contemporary intervention devices reported that new generation bioabsorbable polymers biolimus-eluting stents demonstrated superior clinical outcomes when compared with older generation DES (59). In addition, a pooled analysis yielded similar conclusions (60). Unfortunately, our findings failed to validate the superiority of New-DES for Old-SES in *de novo* lesions of small arteries despite the New-DES ranking highest but do so for PES specifically. It is also important to note that the comparison between the Old-SES and New-DES was derived only from an indirect comparison, and there was no direct comparison of the results to prove the inferiority or superiority of both, and this limited statistical power may be the reason for the lack of statistical difference between them. Therefore, head-to-head studies are needed in the future to investigate whether differences are available between Old-SES and New-DES.

Another finding is that there is no association with the risk of all-cause mortality and MI, regardless of the intervention device used. It has been demonstrated that the type of intervention devices was independent of the incidence of MI in the long-term follow-up (61). Indeed, in our network meta-analysis, although PCB and DES without PES significantly reduced the risk of MACE and TLR, this benefit did not extend to all-cause mortality and MI, which requires additional trials to explore the contributing factors affecting them.

## Limitations

Firstly, the inconsistency of the patient population in the included studies may lead to instability of the results. Secondly, varying follow-up times may result in the benefits of different intervention strategies for certain clinical outcomes not being apparent, but for our study, the longest follow-up time was selected. Thirdly, the definition of the primary outcome was not uniform across the included trials, but this limitation is frequently encountered in all meta-analyses. In addition, small coronary arteries were defined as ≤3 mm in diameter in this network meta-analysis, but the cut-offs used to define small vessels varied among the included studies, and so there is a need for further studies to compare the effectiveness of different intervention strategies in “truly” small vessels. Finally, despite the inclusion of sensitivity analyses, with the exception of BMS vs. BA, there are limited studies directly comparing intervention devices.

## Conclusion

This network meta-analysis provides evidence that when compared with new-generation DES, DCB is associated with comparable outcomes in treating *de novo* lesions of small coronary arteries, suggesting that DCB may be a favorable alternative intervention strategy for small vessels.

## Data availability statement

The original contributions presented in this study are included in the article/Supplementary material, further inquiries can be directed to the corresponding author.

## Author contributions

Y-JZ conceptualized and designed the study. W-RM, KC, and C-SN collected the data, conducted the analyses, and drafted the manuscript. Y-JZ, JI, and CB revised the manuscript. Y-XZ, SC, Y-WC, and X-YW performed the data extraction. All authors read and approved the final manuscript.
