# Supplementary material for: Clinical outcomes of percutaneous coronary intervention for de novo lesions in small coronary arteries: A systematic review and network meta-analysis
Source: Front Cardiovasc Med. 2022 Nov 14;9:1017833. doi: 10.3389/fcvm.2022.1017833 (PMC9702822; doi:10.3389/fcvm.2022.1017833)

**Supplementary Table 1.** Search strategy on PubMed, Embase and Cochrane

| **PubMed** | |
| --- | --- |
| #1 | "percutaneous coronary intervention"[Title/Abstract] OR "PCI"[Title/Abstract] OR "coronary intervention"[Title/Abstract] |
| #2 | "acute coronary syndrome"[Title/Abstract] OR "ACS"[Title/Abstract] OR "stable angina"[Title/Abstract] OR "myocardial infarction"[Title/Abstract] OR "NSTEMI"[Title/Abstract] OR "STEMI"[Title/Abstract] OR "coronary disease"[Title/Abstract] OR "coronary artery disease"[Title/Abstract] |
| #3 | #1 OR #2 |
| #4 | "small vessel*"[Title/Abstract] OR "small coronary arter*"[Title/Abstract] OR "small coronary vessel*"[Title/Abstract] OR "small vessel disease"[Title/Abstract] |
| #5 | #3 AND #4 |
| #6 | "drug eluting stent*"[Title/Abstract] OR "drug coated stent*"[Title/Abstract] OR "drug eluting stent*"[Title/Abstract] OR "stent*"[Title/Abstract] OR "eluting stent*"[Title/Abstract] OR "biodegradable polymer"[Title/Abstract] |
| #7 | "drug eluting balloon*"[Title/Abstract] OR "drug coated balloon*"[Title/Abstract] OR "paclitaxel coated balloon"[Title/Abstract] OR "paclitaxel eluting balloon"[Title/Abstract] |
| #8 | "bare metal"[Title/Abstract] OR "balloon angioplasty"[Title/Abstract] OR "coronary angioplast*"[Title/Abstract] OR "PTCA"[Title/Abstract] OR "percutaneous transluminal coronary angioplasty"[Title/Abstract] |
| #9 | #6 OR #7 OR #8 |
| #10 | "randomized controlled trial"[Publication Type] OR "randomized trial"[Title/Abstract] OR "randomized study"[Title/Abstract] OR "randomized"[Title/Abstract] OR "trial"[Title/Abstract] |
| #11 | #5 AND #9 AND #10 |

| **Embase** | |
| --- | --- |
| #1 | coronary AND ('artery'/exp OR artery) AND ('disease'/exp OR disease) |
| #2 | small AND coronary AND ('artery'/exp OR artery) |
| #3 | #1 AND #2 |
| #4 | ('drug'/exp OR drug) AND eluting AND ('stent'/exp OR stent) |
| #5 | 'biodegradable polymer'/exp OR 'biodegradable polymer' |
| #6 | 'drug-coated balloon'/exp OR 'drug-coated balloon' |
| #7 | bare AND ('metal'/exp OR metal) AND ('stent'/exp OR stent) |
| #8 | 'percutaneous transluminal angioplasty'/exp OR 'percutaneous transluminal angioplasty' |
| #9 | #4 OR #5 OR #6 OR #7 OR #8 |
| #10 | #3 AND #9 AND ‘randomized controlled trial’ |

| **Cochrane** | |
| --- | --- |
| #1 | ("coronary artery disease"):ti,ab,kw |
| #2 | Small coronary artery |
| #3 | #1 AND #2 |
| #4 | Drug-eluting stent |
| #5 | Bare metal stent |
| #6 | Balloon angioplasty |
| #7 | Drug-coated balloon |
| #8 | #4 OR #5 OR #6 OR #7 |
| #9 | Randomized trial |
| #10 | #3 AND #8 AND #9 |

**Supplementary Table 2. Used devices (DES, DCB, BMS) across the trials.**

| Trial | Interventions | Devices | Type |
| --- | --- | --- | --- |
| Ardissino et al. | SES  BMS | SES (Cypher balloon-expandable stent; Cordis, Miami Lakes, Fla)  BMS (Bx Sonic balloon-expandable stent; Cordis) | Older generation SES  BMS |
| BELLO | DCB  PES | IN.PACT Falcon paclitaxel DCB (Medtronic, Inc., Santa Rosa, California)  PES (Taxus Libertè, Boston Scientific, Boston, Massachusetts) | PCB  PES |
| BESMART | BMS  BA | BeStent Small (Medtronic Inc) | BMS  BA |
| BIO-RISE CHINA | DCB  BA | BCB (Biolimus BA9, Biosensor International, China) | BCB  BA |
| COAST | BMS  BA | Heparin-coated JOSTENT Flex stent | BMS  BA |
| C-SIRIUS | SES  BMS | SES (CypherTM, Cordis, Johnson & Johnson)  BMS (Bx-VELOCITY stent, J&J Cordis, Miami Lakes, Florida) | Older generation SES  BMS |
| De luca et al. | BMS  BA | Bx Velocity; Cross-flex; NIR | BMS  BA |
| E-SIRIUS | SES  BMS | SES (Bx Velocity stents, Cordis, Miami Lakes, FL, USA)  BMS ((Bx Velocity stents, Cordis, Miami Lakes, FL, USA) | Older generation SES  BMS |
| Funatsu et al. | DCB  BA | PCB (SeQuent Please, B.Braun, Berlin, Germany) | PCB  BA |
| Hanekamp et al. | BMS  BA | Tenax stent (Biotronik, GmbH, Berlin, Germany) | BMS  BA |
| ISAR-SMART 3 | SES  PES | SES (CypherTM, Cordis, Johnson & Johnson)  PES (TaxusTM, Boston Scientific) | Older generation SES  PES |
| Kinsara et al. | BMS  BA | Heparin coated Jo Stents (JOMED, Rangendingen, Germany) | BMS  BA |
| LASMAL I | BMS  BA | BiodivYsio SV stent (Abbott Vascular, Abbott Park, IL) | BMS  BA |
| LASMAL II | BMS  BA | Biodivisio SV stent (Abbott Vascular Inc, Redwood City, Calif) | BMS  BA |
| Park et al. | BMS  BA | NIR stent (Boston Scientific Corporation, Boston, USA) | BMS  BA |
| PICCOLETO | DCB  PES | Dior PCB (Eurocor, Bonn, Germany)  Taxus Libertè DES (Boston Scientific Corporation, Natick, MA, USA) | PCB  PES |
| PICCOLETO II | DCB  EES | Elutax SV/Emperor  EES (Xience, Abbott Vascular, Santa Clara, CA, USA) | PCB  New generation DES |
| RESTORE | DCB  ZES | Restore DCB (Cardionovum, Bonn, Germany)  RESOLUTE Integrity ZES (Medtronic Inc., Santa Rosa, CA, USA) | PCB  New generation DES |
| SISA | BMS  BA | BeStent Artist (Medtronic Vascular) | BMS  BA |
| SISCA | BMS  BA | BeStent Small (Medtronic Inc) | BMS  BA |
| SPIRIT III | EES  PES | XIENCE V (Abbott Vascular, CA, USA)  TAXUS Express (Boston Scientific Corporation, Natick, MA, USA) | New generation DES  PES |
| STRESS | BMS  BA | Palmaz-Schatz stents | BMS  BA |
| TAXUS V subgroup | PES  BMS | PES (Taxus Libertè, Boston Scientific, Boston, Massachusetts)  BMS (Boston Scientific, Boston, Massachusetts) | PES  BMS |

BA, balloon angioplasty; BMS, bare-metal stent; DCB, drug-coated balloon; EES, everolimus-generation drug-eluting stent; PES, paclitaxel-eluting stent; SES, sirolimus-eluting stent; ZES, zotarolimus-eluting stent.

**Supplementary Table 3.** Randomized controlled trials included in the network meta-analysis.

| Trial | Interventions | The definition of MACE | The result of MACE |
| --- | --- | --- | --- |
| Ardissino et al. | SES/BMS | Death, Q- or non-Q-wave MI, TVR | SES superior to BMS |
| BELLO | PCB/PES | Death, Q- or non-Q-wave MI, TVR | PCB noninferior to PES |
| BESMART | BMS/BA | Death, MI, repeat revascularization | BMS superior to BA |
| BIO-RISE CHINA | BCB/BA | Death, MI, repeat revascularization | BCB noninferior to BA |
| COAST | BMS/BA | - | - |
| C-SIRIUS | SES/BMS | Death, MI, TLR, emergent CABG | SES superior to BMS |
| De luca et al. | BMS/BA | No definition | No significant differences between BMS and BA |
| E-SIRIUS | SES/BMS | Death, MI, TLR, CABG | SES superior to BMS |
| Funatsu et al. | PCB/BA | Cardiac death, MI, TVR | PCB noninferior to BA |
| Hanekamp et al. | BMS/BA | Death, MI, TVR | No significant differences between BMS and BA |
| ISAR-SMART 3 | SES/PES | Death, MI | No significant differences between SES and PES |
| Kinsara et al. | BMS/BA | - | - |
| LASMAL I | BMS/BA | Death, MI, repeat revascularization | BMS superior to BA |
| LASMAL II | BMS/BA | Death, MI, repeat revascularization | No significant differences between BMS and BA |
| Park et al. | BMS/BA | - | - |
| PICCOLETO | PCB/PES | MACE, Q-wave MI, TLR | PCB noninferior to PES |
| PICCOLETO II | PCB/EES | Cardiac death, MI, TLR | PCB noninferior to EES |
| RESTORE | PCB/ZES | Cardiac death, MI, TLR | PCB noninferior to ZES |
| SISA | BMS/BA | Death, MI, TVR | No significant differences between BMS and BA |
| SISCA | BMS/BA | Cardiac death, AMI, TLR, TVR | BMS superior to BA |
| SPIRIT III | EES/PES | Cardiac death, MI, TLR | EES superior to PES |
| STRESS | BMS/BA | Death, MI, TVR | BMS superior to BA |
| TAXUS V subgroup | PES/BMS | No definition | No significant differences between PES and BMS |

BA, balloon angioplasty; BMS, bare-metal stent; DCB, drug-coated balloon; EES, everolimus-generation drug-eluting stent; MACE, major adverse cardiac events; MI, myocardial infarction; PES, paclitaxel-eluting stent; SES, sirolimus-eluting stent; TLR, target lesion revascularization; TVR, target vessel revascularization; ZES, zotarolimus-eluting stent.

**Supplementary Table 4.** The primary network meta-analysis estimates of all-cause mortality and MI

| A |  |  |  |  |  |  |
| --- | --- | --- | --- | --- | --- | --- |
| All-cause mortality | | | | | | |
| BCB | 4.53 (0.12,171.20) | 5.20 (0.25,106.92) | 5.29 (0.18,152.65) | 5.88 (0.28,124.93) | 7.56 (0.15,377.22) | 7.32 (0.24,221.40) |
| 0.22 (0.01,8.34) | PCB | 1.15 (0.15,8.57) | 1.17 (0.17,7.97) | 1.30 (0.18,9.46) | 1.67 (0.27,10.14) | 1.62 (0.35,7.36) |
| 0.19 (0.01,3.96) | 0.87 (0.12,6.52) | BA | 1.02 (0.23,4.42) | 1.13 (0.72,1.77) | 1.46 (0.12,17.34) | 1.41 (0.29,6.80) |
| 0.19 (0.01,5.45) | 0.86 (0.13,5.84) | 0.98 (0.23,4.27) | Old-SES | 1.11 (0.27,4.55) | 1.43 (0.13,15.39) | 1.38 (0.38,5.06) |
| 0.17 (0.01,3.62) | 0.77 (0.11,5.63) | 0.88 (0.57,1.38) | 0.90 (0.22,3.69) | BMS | 1.29 (0.11,14.99) | 1.25 (0.27,5.73) |
| 0.13 (0.00,6.60) | 0.60 (0.10,3.64) | 0.69 (0.06,8.19) | 0.70 (0.06,7.54) | 0.78 (0.07,9.05) | New-DES | 0.97 (0.13,7.47) |
| 0.14 (0.00,4.13) | 0.62 (0.14,2.82) | 0.71 (0.15,3.43) | 0.72 (0.20,2.65) | 0.80 (0.17,3.69) | 1.03 (0.13,7.97) | PES |
| B |  |  |  |  |  |  |
| MI | | | | | | |
| BCB | 2.28 (0.17,31.50) | 3.45 (0.38,31.36) | 3.35 (0.32,35.12) | 4.36 (0.25,76.46) | 4.10 (0.36,46.35) | 4.16 (0.47,36.79) |
| 0.44 (0.03,6.03) | PCB | 1.51 (0.36,6.36) | 1.47 (0.36,5.96) | 1.91 (0.51,7.10) | 1.79 (0.60,5.37) | 1.82 (0.42,7.83) |
| 0.29 (0.03,2.64) | 0.66 (0.16,2.79) | BMS | 0.97 (0.44,2.14) | 1.26 (0.20,7.95) | 1.19 (0.43,3.27) | 1.21 (0.85,1.71) |
| 0.30 (0.03,3.12) | 0.68 (0.17,2.77) | 1.03 (0.47,2.26) | Old-SES | 1.30 (0.21,7.92) | 1.22 (0.49,3.07) | 1.24 (0.52,2.97) |
| 0.23 (0.01,4.02) | 0.52 (0.14,1.95) | 0.79 (0.13,4.98) | 0.77 (0.13,4.68) | New-DES | 0.94 (0.19,4.55) | 0.95 (0.15,6.12) |
| 0.24 (0.02,2.76) | 0.56 (0.19,1.67) | 0.84 (0.31,2.31) | 0.82 (0.33,2.05) | 1.06 (0.22,5.14) | PES | 1.01 (0.35,2.93) |
| 0.24 (0.03,2.13) | 0.55 (0.13,2.37) | 0.83 (0.58,1.18) | 0.81 (0.34,1.93) | 1.05 (0.16,6.72) | 0.99 (0.34,2.85) | BA |

BA, balloon angioplasty; BCB, biolimus-coated balloon; BMS, bare-metal stent; MI, myocardial infarction; New-DES, new-generation drug-eluting stent; Old-SES, older generation sirolimus-eluting stent; PCB, paclitaxel-coated balloon; PES, paclitaxel-eluting stent.

**Supplementary Table 5.** The primary network meta-analysis estimates of MACE and TLR

| A | | | |
| --- | --- | --- | --- |
| MACE | | | |
| DES | 1.09 (0.78,1.50) | 2.10 (1.49,2.95) | 2.83 (1.95,4.13) |
| 0.92 (0.66,1.27) | DCB | 1.93 (1.28,2.90) | 2.61 (1.72,3.96) |
| 0.48 (0.34,0.67) | 0.52 (0.34,0.78) | BMS | 1.35 (1.11,1.64) |
| 0.35 (0.24,0.51) | 0.38 (0.25,0.58) | 0.74 (0.61,0.90) | BA |
| B | | | |
| TLR | | | |
| DES | 1.14 (0.78,1.66) | 2.85 (1.93,4.20) | 4.19 (2.75,6.39) |
| 0.88 (0.60,1.28) | DCB | 2.51 (1.54,4.08) | 3.69 (2.25,6.05) |
| 0.35 (0.24,0.52) | 0.40 (0.24,0.65) | BMS | 1.47 (1.21,1.78) |
| 0.24 (0.16,0.36) | 0.27 (0.17,0.45) | 0.68 (0.56,0.83) | BA |

BA, balloon angioplasty; BMS, bare-metal stent; DCB, drug-coated balloon; DES, drug-eluting stent; MACE, major adverse cardiac events; TLR, target lesion revascularization.

**Figure title and legends**

**Supplementary Figure 1.** Risk of bias assessment

**Supplementary Figure 2.** Funnel plots of all clinical outcomes of interest

A), major adverse cardiac events; B), target lesion revascularization; C), all-cause mortality; D), myocardial infarction.

A, balloon angioplasty; B, biolimus-coated balloon; C, bare-metal stent; D, new-generation drug-eluting stent; E, older generation sirolimus-eluting stent; F, paclitaxel-coated balloon; G, paclitaxel-eluting stent.

**Supplementary Figure 3.** Inconsistency plot

**Legend:** Inconsistency plot assuming loop-specific heterogeneity. A τ^2^ value less than 0.1 indicated a very low level of heterogeneity, and a τ^2^ value from 0.1 to 0.5 indicated a reasonable level; a τ^2^ value greater than 0.5 was considered toindicate high heterogeneity. A), all-cause mortality; B), major adverse cardiac events; C), target lesion revascularization.

A, balloon angioplasty; B, biolimus-coated balloon; C, bare-metal stent; D, new-generation drug-eluting stent; E, older generation sirolimus-eluting stent; F, paclitaxel-coated balloon; G, paclitaxel-eluting stent.

**Supplementary Figure 4.** Inconsistency plot

**Legend:** Inconsistency plot assuming loop-specific heterogeneity. A τ2 value less than 0.1 indicated a very low level of heterogeneity, and a τ2 value from 0.1 to 0.5 indicated a reasonable level; a τ2 value greater than 0.5 was considered toindicate high heterogeneity. A), myocardial infarction; B), major adverse cardiac events; C), target lesion revascularization.

A, balloon angioplasty; B, biolimus-coated balloon; C, bare-metal stent; D, new-generation drug-eluting stent; E, older generation sirolimus-eluting stent; F, paclitaxel-coated balloon; G, paclitaxel-eluting stent.

**Supplementary Fig. 1** Risk of bias assessment


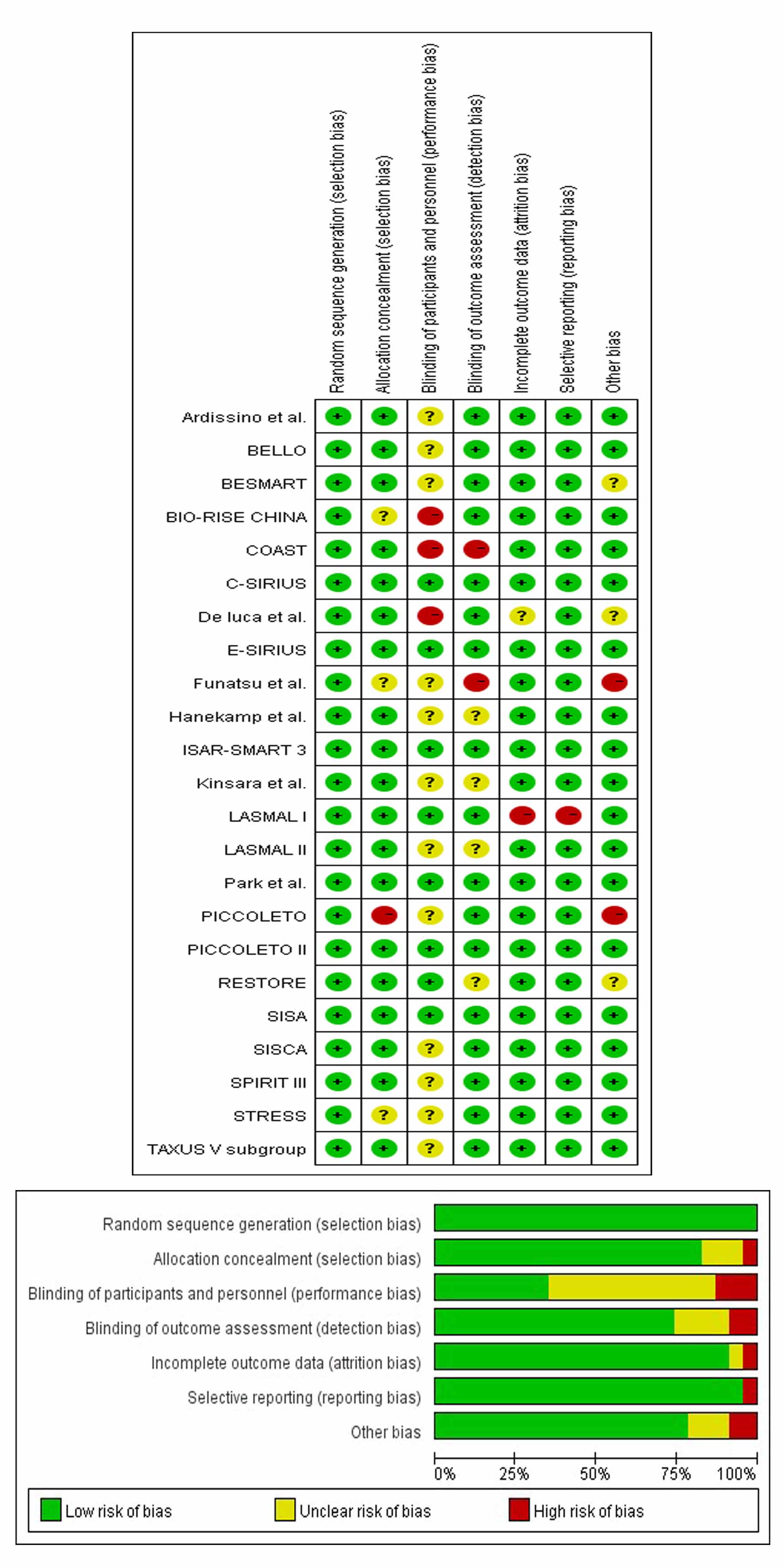


**Supplementary Fig. 2** Funnel plots of all clinical outcomes of interest


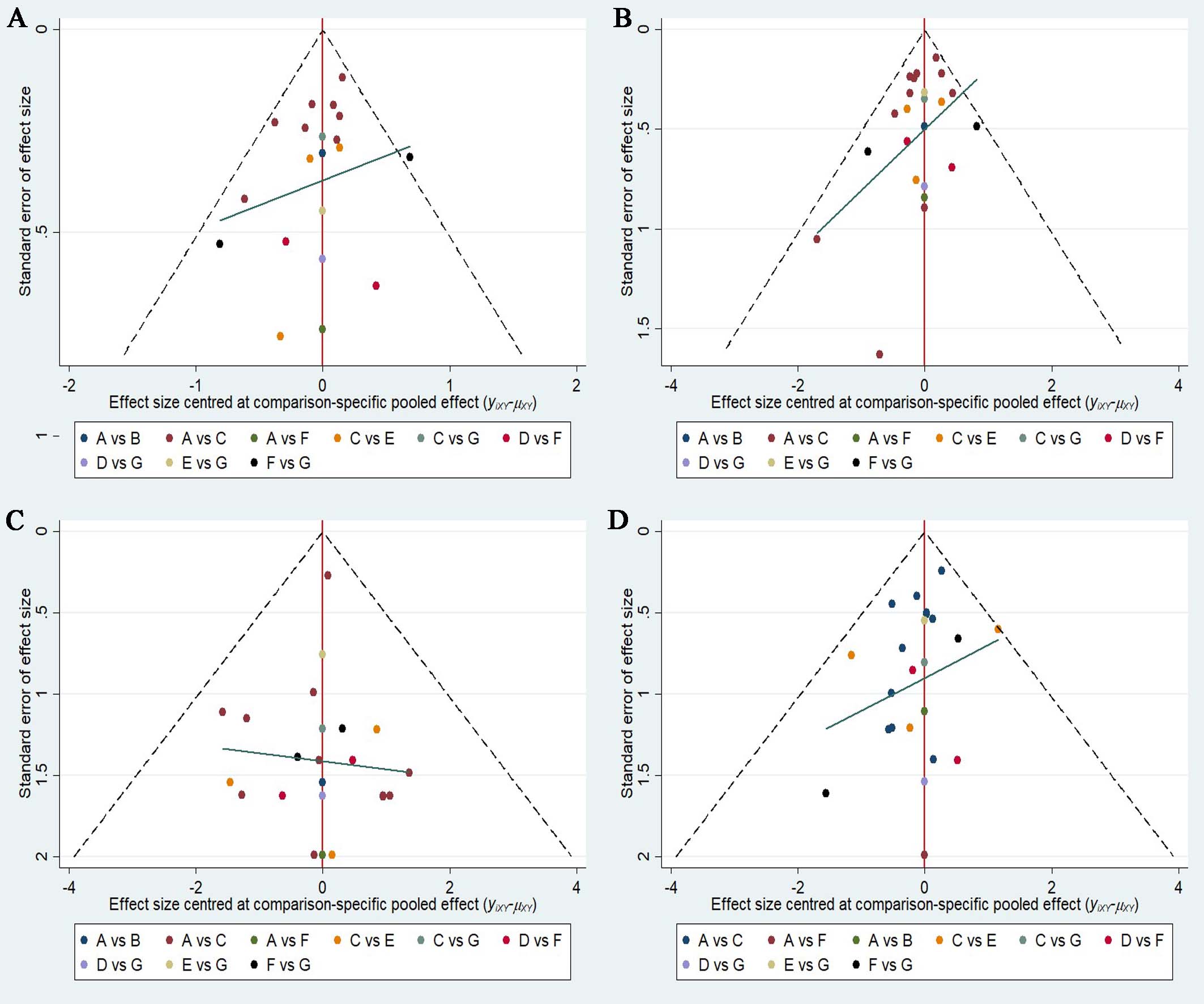


**Supplementary Fig. 3** Inconsistency plot


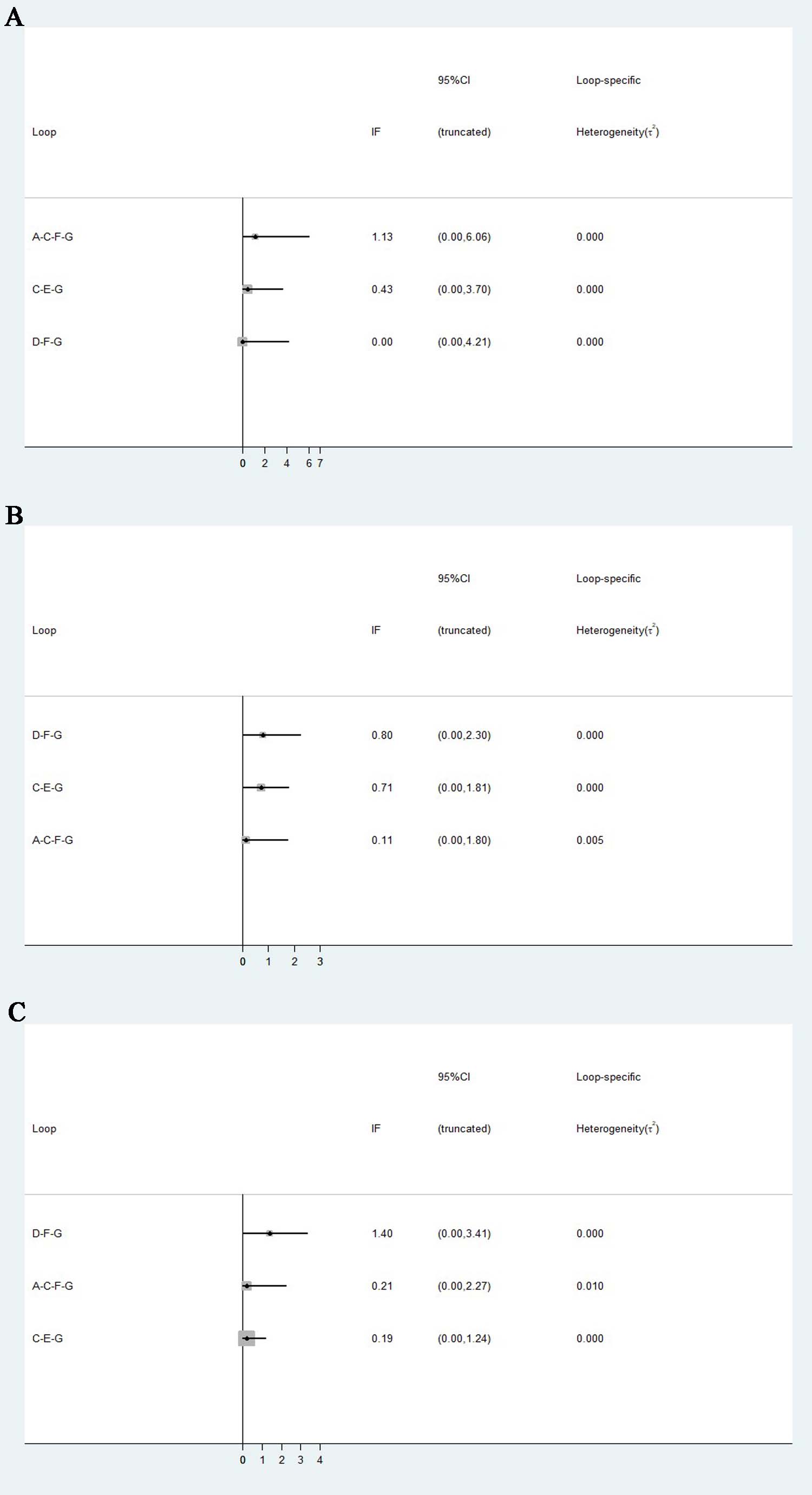


**Supplementary Fig. 4** Inconsistency plot


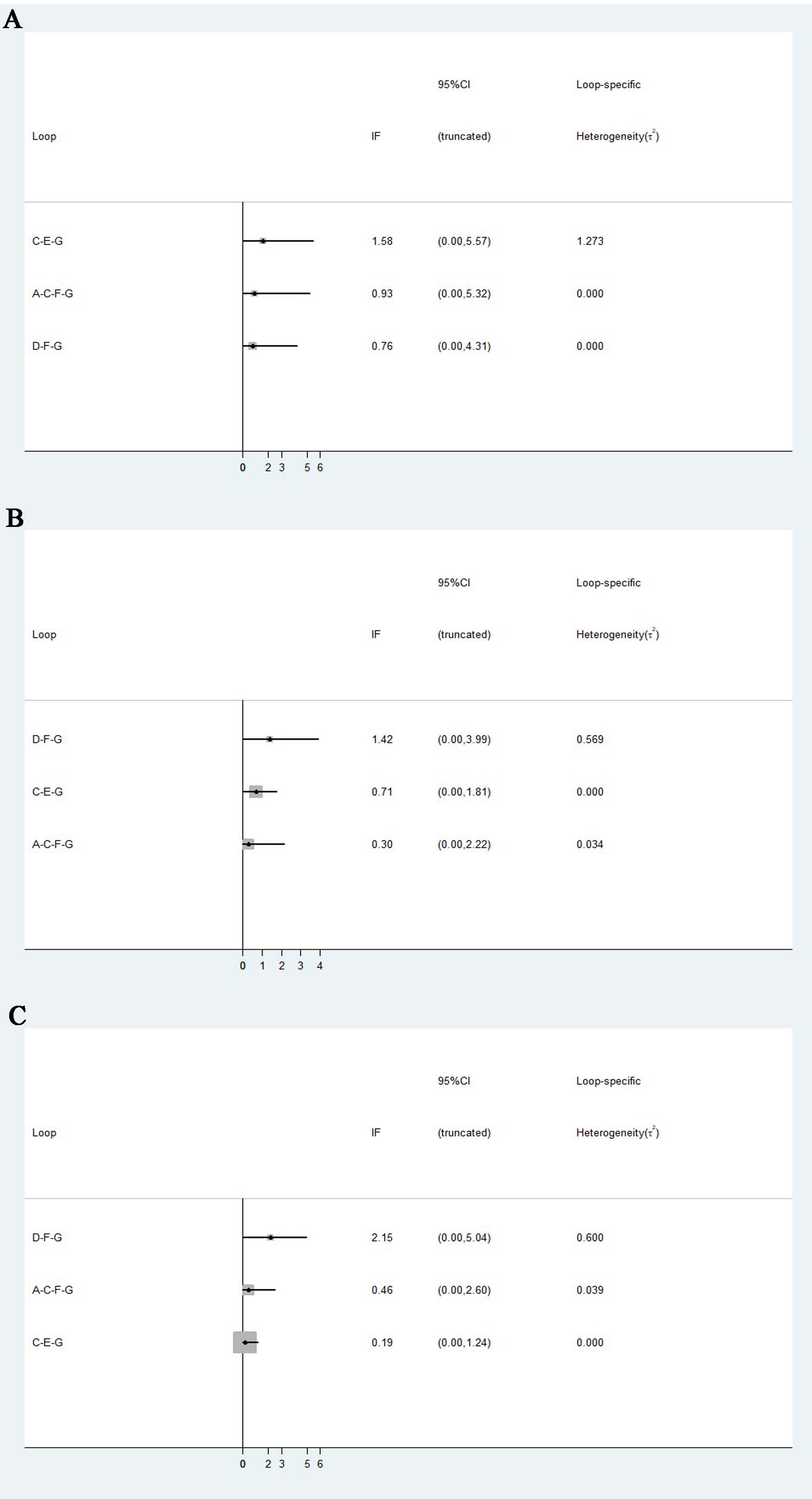

Supplement: Supplementary file 1 [file Data_Sheet_1.doc]
